# Dogs with prior experience of a task still overimitate their caregiver

**DOI:** 10.1038/s41598-024-70700-3

**Published:** 2024-09-06

**Authors:** Louise Mackie, Ludwig Huber

**Affiliations:** Department of Interdisciplinary Life Sciences, Comparative Cognition, Messerli Research Institute, University of Veterinary Medicine Vienna, Vienna, Austria

**Keywords:** Domestic dogs, Prior experience, Overimitation, Social learning, Causal understanding, Psychology, Zoology, Animal behaviour, Human behaviour

## Abstract

Domestic dogs have been shown to copy their caregiver’s actions, including ones which are causally-irrelevant to a physical goal—a behaviour called “overimitation”. In a new overimitation task with a non-food reward, this study investigated “causal misunderstanding”—falsely assuming causally-irrelevant actions to have functional relevancy—as an explanation for dog overimitation (N = 81). By providing dogs with prior experience of the task to learn about the consequences of its irrelevant box-stepping and relevant bucket-opening action to obtain a toy-ball, we tested whether and when dogs would copy their caregiver’s irrelevant-action demonstrations. Dogs with and without prior experience were compared to a third (control) group of dogs, who had neither prior experience nor caregiver demonstrations of the task. Results revealed that the timing of overimitation, rather than its frequency, was closely related to dogs' prior experience: dogs with prior experience attended to their reward first, then interacted with the irrelevant box later (“post-goal overimitation”), while dogs without prior experience first interacted with the irrelevant box (“pre-goal overimitation”). Our results suggest that, when action consequences are understood, dogs are overimitating for a secondary social goal that is clearly distinct from the task goal of obtaining a physical reward.

## Introduction

Unlike many animal species, domestic dogs (*Canis lupus familiaris*) have long had an ecological niche of living alongside humans within their societies (e.g.^1^). This has allowed dogs to develop impressive human-like sociocognitive abilities, such as the ability to watch and learn from human actions. For example, when a human demonstrator shows a dog one of two solutions to a novel task to find food, dogs have a tendency to copy the solution demonstrated over finding their own solution to the task (e.g.^2,3^). Even in the absence of food rewards, dog puppies match a human’s action more than wolf pups or kittens, which suggests that dogs have a predisposition to socially learn from and pay close attention to humans^4^. Dogs are also known to excel in do-as-I-do tasks, meaning that they can be trained to copy both familiar and completely novel actions of humans on command (i.e.^5–7^). Being able to learn from human actions not only provides dogs with ways to obtain knowledge of their environment, such as knowledge that opening the kitchen cupboard gives access to biscuits, but it may also provide them with chances to affiliate with their human partners.

Dogs have been shown to copy even causally-irrelevant actions of humans^8^, but also see^9^, and more so when that human is their closely-bonded caregiver^10,11^. These are actions which have no functional significance towards reaching a physical goal in a task, such as a food reward, while causally-relevant actions do. Although this behaviour has been commonly referred to as “overimitation”^12^, it should be noted that perfect bodily imitation of irrelevant actions is not expected. Simply engaging with an irrelevant mechanism (like putting a stick in an irrelevant hole) can suffice for overimitation, as the precise manner of how irrelevant actions are performed compared to how they were demonstrated is not typically measured (see^13^). In the human developmental literature, overimitation has been said to result from “causal distortions” or “causal misunderstandings” of demonstrated actions: when individuals automatically, and falsely, codes all observed actions as causally relevant to a task’s goal^14^. Accordingly, only later do human children begin to question the causal significance of actions towards a goal. Schleihauf and Hoehl^15^ explain that individuals deliberately consider whether to copy someone’s irrelevant actions once they have clear understanding of their causal *in*significance—once prior causal misunderstandings from the (initial) automatic overimitation process are elucidated. In this case, overimitation becomes socially selective. It is influenced by social factors including: whether the demonstrator behaved prosocially before the task by helping another person^16^, was an in-group member ^17^, or shared the same first language^18^. Taken together, the human overimitation literature has clearly established multiple reasons and processes behind overimitation’s occurrence (^19^ for a review). However, why exactly dogs overimitate, be it from a simple kind of causal misunderstanding or pure affiliation, remains unclear.

In dogs, overimitation does not occur as frequently as it does in humans. Around 49% of dogs touched at least one coloured dot on the wall in Huber et al.’s^8^ task, and 59% of dogs used an irrelevant lever by trial four in Johnston et al.’s^9^ task. Yet, overimitation frequencies were still at 70–80% after six trials for human children in the classic Horner and Whiten^20^ task. Even though dogs may not be overimitating strictly in the same way as humans, they do seem to have some social motivations behind their behaviour. For example, dogs have overimitated their caregiver more than a stranger^10^, have scored highly for both overimitation and caregiver-relationship tasks^11^, and have overimitated most often *after* already obtaining their food reward in a task^21^. However, causal misunderstanding may still be contributing to overimitation in dogs.

Dogs are famously “poor” at causal understanding—an important skill if one is to distinguish between irrelevant and relevant actions. By the term “causal understanding”, we henceforth refer to a general associative interpretation for dogs; having the knowledge of an effect, rather than knowing or questioning why it occurs. That is, whether dogs can *know* that B follows A, rather than dogs can know *why* A causes B. The idea that domestication led to dogs losing some ability to make causal connections came from a study that directly compared the physical and social cognition of dogs and great apes^22^. Bräuer et al. found that dogs performed worse on tasks that required forming connections from physical cues, but better on tasks that required the utilisation of social cues like human gestures. Their results established the “social dog, causal ape” hypothesis, which would support causal misunderstanding as an explanation for overimitation in dogs. That dogs are copying a demonstrator’s causally-irrelevant actions because they fail to understand that the irrelevant actions do not relate to a task’s reward.

In the previous overimitation tasks, dogs still used an irrelevant lever in their fourth trial of Johnston et al.’s^9^ study, and dogs also still touched the irrelevant dots in trial four of Mackie and Huber’s^21^ study. This suggests that these dogs either did not learn of the action’s (causal) unnecessity over trials, or the dogs did learn it, yet copied the irrelevant action anyway for reasons unrelated to getting the food reward. Individual differences in the problem-solving behaviour of dogs is a still-growing field, but by manipulating what dogs have (or have not) already learned about our task we can thus address the particular issue involving their understanding of action relevancy in overimitation. Regarding causal transparency of task actions, the dot-touching task had an advantage: Its irrelevant action was spatially separated and physically disconnected from the food reward, meaning that it was less likely that dogs were misunderstanding its relevancy to the goal. However, since food is often replenishable, it cannot be excluded that dogs could have formed expectations that touching the dot(s) would somehow produce more food in the task’s food chamber through hidden causations. Therefore, the two explanations still remain for these dogs who overimitated,either there were elements of causal misunderstanding, or these dogs understood that the irrelevant action did not lead to the food but they still overimitated for “non-instrumental” reasons—reasons distinct from the physical pathway of producing the reward. Causal misunderstanding of the irrelevant action needs to be isolated and removed before we can proceed with the investigation of dogs’ social motivations to overimitate.

The present study aimed to address this kind of causal misunderstanding and overimitation in dogs directly, by providing dogs with prior experience of the target irrelevant and relevant actions in a new overimitation task with a toy-ball reward. Prior experienced dogs already learned of the consequences of the (spatially separated) irrelevant actions and relevant actions before the task, thus any potential causal misunderstandings of how to get their ball reward were omitted. The non-food reward also persisted in the room once the dogs managed to obtain it from the relevant action, therefore, any “*post-goal”* overimitation (overimitation after the goal) in our new task could not be related to trying to obtain more rewards through hidden causations—their reward was available for interaction within the remaining time of their trial. Additionally, post-goal overimitation previously observed in dogs and said to be unrelated to the task's physical goal^21^, may have been influenced by a recency effect, since task relevant actions are usually demonstrated last (i.e.^9^ and^8^ tasks). Therefore, we also included (caregiver) demonstrations for each trial in which the irrelevant action was shown before *and* after the relevant action to avoid any recency effect on action order, i.e., from observing the ball reward in the causally-relevant container immediately before being released for a trial.

We predicted that if dogs were simply having some kind of causal misunderstandings, then those with prior experience would attend to the irrelevant action less than those without. In humans, having prior experience made children more likely to ignore irrelevant actions that were later demonstrated to them^23^. Similarly, children who observed an efficient demonstration first, and an inefficient one second, tended to not adopt the new irrelevant actions^24^. So if dogs are overimitating *entirely* due to some sort of causal misunderstanding, then those with prior experience should not overimitate at all in this (new, causally transparent) task. Additionally, since Mackie and Huber^21^ found an effect of action order, we tested if the timing that which the dog attended to the irrelevant action, before or after reaching the goal of the relevant action, was also influenced by prior experience. Lastly, we included a no-demonstration control group in this study, in which dogs had neither prior experience nor caregiver demonstrations. By comparing our two experimental groups’ performance (dogs with and dogs without prior experience) to a control we could confirm that our levels of irrelevant- and relevant-action copying in this new task were actually a result of the social demonstrations, and not individual learning.

## Methods

### Ethical statement

The University of Veterinary Medicine Vienna’s ethical committee approved this study and its procedures, in agreement with good scientific practice and national legislation guidelines (ref: ETK-029/02/2023). This study is reported in accordance with ARRIVE guidelines. Dogs in this study engaged in a non-invasive problem-solving task to obtain a toy reward (ball) at the Clever Dog Lab in Vienna, Austria.

This study’s design, methods, and (most of the) main analyses were pre-registered on As Predicted, which can be viewed via the following link: https://aspredicted.org/Y73_VFL.

### Subjects

The final sample of this study contained 81 family dogs (48 female, 33 male) after four dogs were excluded for a lack of ball motivation, which was a pre-requisite of the study (85 tested dogs in total, plus 14 pilot dogs). Other pre-requisites were: to be over 1-year-old and to have had at least 3 months with the caregiver. Eighteen of the first dogs (20%) were randomly assigned to one of three groups (with prior experience, without prior experience, or the control), while the remaining dogs were assigned to balance the groups for; sex, age, breed type, Clever Dog Lab experience, and previous overimitation task experience. Our prior-experience group had 25 dogs (mean 5.06 years old, 15 female), our no-prior-experience group had 28 dogs (mean 4.86 years old, 16 female), and our control group had 28 dogs (mean 5.18 years old, 17 female).

The Participant List in the Supplementary Materials contains all individual dog information, such as that mentioned above.

Dogs and their caregivers were recruited through the Clever Dog Lab database and website, social media, and from dog parks.

### Design and materials

#### Design

The present study had a mixed design where we manipulated whether or not dogs had prior experience of our box-stepping overimitation task (Table 1). Our two experimental groups (with or without prior experience) and control group were between-subject and our four test trials were within-subject (as in^21^). Only the experimenter was aware of the group allocations during testing.

**Table 1:** The different dog groups and their procedures for the testing session.

Groups (between-subject)	(1) 1-min habituation	(2a) Prior experience phase	(2b) Item habituation phase	(3) Ball-motivation phase	(4) Box-stepping overimitation task (4 trials within-subject)
No prior experience	×		×	×	×
Prior experience	×	×		×	×
Baseline (control)	×		×	×	× (no demonstrations)

There were four phases of the testing session: (1) a 1-min habituation phase for the testing room, either (2a) a prior experience phase or (2b) an item habituation phase (depending on condition), (3) a ball-motivation phase, and (4) the main box-stepping overimitation task, which included four trials with task caregiver demonstrations (each trial). No food rewards were used during testing (only a toy-ball reward). Before and between each phase, the experimenter explained the upcoming procedure to the caregiver outside of the testing room, allowing for a short (2–3-min) break, any questions, and time for the experimenter to set up and rearrange the testing room.

The whole session took around 20 min and was recorded on two video cameras (Panasonic HC-V777, http://panasonic.com) and one ceiling camera. Video outputs were framed together in an .mp4 video file and uploaded to an in-house Loopy server (http://loopbio.com/loopy, loopbio, Vienna, Austria) for secure data storage.

#### Materials

**Fig. 1:**
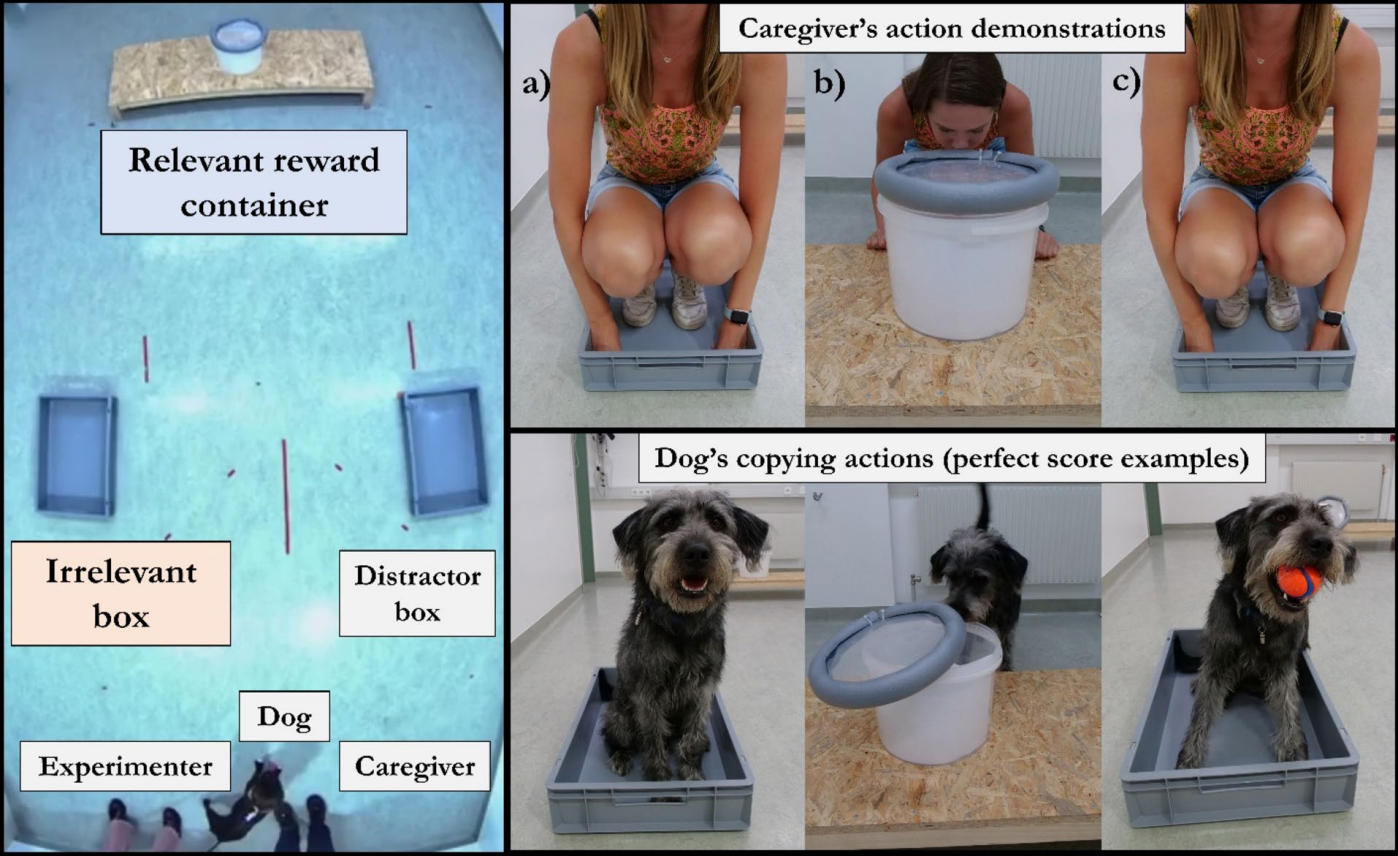
The box-stepping overimitation task. The left picture depicts the task and its starting positions for the caregiver and dog (birds-eye view). The right pictures are the task’s actions: (**a**) causally-irrelevant box-stepping (before), (**b**) causally-relevant bucket-opening to obtain the ball reward, and (**c**) causally-irrelevant box-stepping (after). Example perfect scores of "4" for each action are depicted by a model caregiver and a model dog (Filou, male).

The box-stepping overimitation task required few items: two open-top boxes (12 cm height, 40 cm width, 60 cm length), one transparent bucket and lid (22.2 cm height 25.7 cm diameter), an A4 piece of paper to obscure the ball from the starting position, a platform (17 cm height, 50 cm width, 149.5 cm length) to stabilise the bucket, and a toy ball (6 cm diameter) for the task reward. The boxes were placed 140 cm apart and 60 cm from the walls, and around 130 cm away from both the dog and the platform (Fig. 1). All items were disinfected and wiped down by the experimenter before each new testing session (and the A4 paper in the bucket was replaced with a fresh one).

An instructional video was created to aid the caregiver in performing their task demonstrations. It showed the demonstration’s three actions being demonstrated by the experimenter with enthusiasm and in a dog-like manner.

### Session procedure

#### 1-min habituation phase

Once caregivers were introduced to the Clever Dog Lab by the experimenter and signed their consent forms, the session began by entering the green testing room (6.0 × 3.3 m). For habituation, dogs (N = 81) were allowed to freely roam the room off-leash for 1-min with the caregiver and experimenter present in the room. After 1 min, the caregiver and dog (on-leash) left the room while the experimenter prepared the item set up for the next phase (2a or 2b).

#### Prior experience phase

Dogs in the prior experience group (N = 25) experienced the box-stepping overimitation task’s goal-irrelevant and relevant actions, to learn of their ("causal") consequences, before the task trials.

The dog entered the testing room on-leash with the caregiver and the experimenter. The room's set up was the same as the main task (Fig. 1). From the room’s door, dogs were either first guided to the boxes or the bucket (order counterbalanced) to experience the corresponding action. For the *relevant action*, the dog was led to the front of the task’s platform and encouraged to push open the bucket lid themselves, in order to take the ball from within the bucket. For example, the experimenter and/or caregiver tapped on the bucket lid while saying “where’s the ball?” until the dog had obtained the ball from the bucket. Once the dog had successfully obtained the ball from the front of the platform, the dog was guided to the back of the platform to repeat the same action. After the dog had gained its prior experience of the relevant-action (twice), the dog, the caregiver, and the experimenter then returned to the room’s door to reset—so that the action experiences were not in one continuous sequence. Next, the dog either gained prior experience of the other action or left the room (depending on order). For the *irrelevant action*, the experimenter, caregiver and dog walked towards one of the two open-top boxes (order counterbalanced). Together, the experimenter and caregiver encouraged the dog to step into the box for its prior experience of the irrelevant action. For example, the caregiver pointed at the box or stepped in the box in front of the dog. Once the dog had stepped into the box with all four paws it was guided back to the door of the room before experiencing the irrelevant action at the second box. Now with experience of both the irrelevant and relevant actions (twice each), the dog left the room with its caregiver while the experimenter remained to prepare the next phase of the session (ball-motivation phase).

#### Item habituation phase

Dogs in the no-prior experience group (N = 28) and the control group (N = 28) did not experience 2a), the prior experience phase, but instead experienced a simple item habituation phase (2b). These dogs were walked around the room to match the habituation that the prior experience group gained of the items and room, while also providing the dogs with equal visual information of the reward’s location (since the ball is obscured from the starting position of the main task). This way, any task performance differences between groups could not be explained by item habituation, but rather the experiences of the actions themselves.

For this phase, the dog entered the testing room (set-up with task items) on-leash with the caregiver and the experimenter. The dog was guided towards and past the task’s items, simply for visual observation. The dog either first approached the bucket or the boxes (order counterbalanced), and between each item, the dog was walked back to the room’s door. After the dog had viewed the reward bucket from the front and the back, and the two boxes from the side, the caregiver and dog left the room on-leash while the experimenter rearranged the room for the next phase (ball-motivation phase).

#### Ball-motivation phase

To ensure that dogs were ball-motivated, and were warmed-up for the main task, all dogs (N = 81) played a game of fetch with their caregiver. The room was cleared of items and the ball reward was given to the caregiver. The experimenter instructed the caregiver to play a light game of fetch with their dog, as they normally would at home. Given that the room may have been slippery to the dog, the caregiver was told to gently and slowly roll or throw the ball. Two out of 81 dogs used their own ball in their testing session for motivational purposes. The ball-motivation phase lasted for 1-min and thirty seconds. Once finished, the caregiver returned the ball to the experimenter before leaving the room with their dog. The experimenter rinsed the ball and prepared the room for the box-stepping overimitation task.

#### The box-stepping overimitation task

Finally, all dogs in the prior experience (N = 25) and no-prior experience groups (N = 28) participated in four 1-min trials, each with caregiver demonstrations, of our box-stepping overimitation task. Control dogs (N = 28) did not receive the four caregiver demonstrations, but had four 1-min trials to independently solve the task. The dog successfully solved the task if it opened the bucket (relevant action) to access the ball reward. The irrelevant action was not necessary to solve this task. A video example of a caregiver demonstration and dog trial is in the Supplementary Materials.

#### *Task demonstration*

The caregiver watched an instructional video with the experimenter to learn how to conduct the task’s demonstration. If the caregiver did not speak English, there were written instructions in German available [Supplementary Materials]. The caregiver practiced the demonstration in the room out-of-sight of their dog. When ready, the dog, caregiver, and experimenter entered the room for the first demonstration and trial. With the dog held on-leash by the experimenter next to the room’s door, the caregiver began the demonstration from the starting position next to the dog (Fig. 1). He/she walked towards the assigned irrelevant box (left or right, counterbalanced), faced the dog, made eye contact and called its name, and then stepped into the box crouching on ‘all fours’ (*irrelevant action (before)*). This dog-like manner was to ensure that the demonstrated actions could be copied by the dog. Next, the demonstrator walked behind the platform and pushed open the lid of the bucket with his/her nose while crouched down facing the dog (*relevant action*). He/she lifted the ball from the bucket to show the dog the reward, then returned the ball and lid to the original place. Finally, the demonstrator stepped into the (same) irrelevant box to perform the irrelevant action once again (*irrelevant action (after)*). The other box served as only a distractor object; interactions with it were considered to be exploration and were not counted towards irrelevant-copying scores. Actions were only demonstrated once the dog was making eye-contact with the caregiver to ensure attention. Control dogs waited by the door for 30 s with the caregiver and experimenter instead of watching a demonstration.

#### *Task trial*

After the task demonstration (for the prior experience and no-prior experience groups), the caregiver stood by the door and the dog was unleashed to begin its 1-min trial to solve the task on its own. During each trial, the caregiver was instructed to stand quiet and still, and to only offer encouragement if necessary (as in^25^). For example, the caregiver could repeat the “OK” release command if the dog was waiting by the owner. Otherwise, the caregiver was told neither to point nor physically aid the dog to complete the task. The 1-min trial continued even if the dog obtained the ball reward. At the end of each trial, if the dog obtained the ball (solved the task) the caregiver praised the dog and gently threw or rolled the ball in the room (away from the items). If the dog did not obtain the ball during its trial, it remained in the bucket. The dog was then leashed and taken out the room by the caregiver. To prepare for the next trial, the experimenter rebaited the bucket and reset the items into their original places (if necessary). After the final (fourth) trial, dogs were praised and a photo was taken for their study certificate.

After the box-stepping overimitation task, the testing session was over. The caregiver was verbally debriefed about the study, and given the chance to ask any further questions. The caregiver also received an A4 certificate with the photo of their dog for their participation.

### Behavioural coding and data analysis

#### Behavioural coding

Dog behaviours of interest were the irrelevant- and relevant-action copying scores from the box-stepping overimitation task (Table 2 for behavioural descriptions of scores). Irrelevant-action scores were coded for both “before” and “after” achieving the task goal (scoring 3 + in the relevant action). If a dog did not achieve the goal in a trial, “before” was still scored and “after” was scored as 0.

**Table 2:** The dog behaviours of interest and their descriptions for coding: copying accuracy score (irrelevant and relevant actions).

Copying accuracy score	Irrelevant action description	Relevant action description
0	No approach to (correct) irrelevant box	No approach to reward container
1	Approach to (correct) irrelevant box (dog walks towards/next to to the box while looking at the box)	Approach to the reward container (dog walks towards/next to the container while looking at the container)
2	Touching of (correct) irrelevant box (with paw or nose)	Touching of the reward container (with paw or nose)
3	Stepping into (correct) irrelevant box (with up to three paws)	Pushing off the lid of the reward container, efficiently from the front of the platform (to obtain the ball)
4	Stepping into (correct) irrelevant box (with all four paws—matching the demonstration)	Pushing off the reward container’s lid, inefficiently from the back of the platform (to obtain the ball—matching the demonstration)

The experimenter (LM) coded all 81 dog videos for these behaviours. Additionally, 20% (16 randomly selected videos) were coded by KG, an external and naïve coder, for coding reliability. KG received a scoring guide (Table 2) and cropped videos, which contained no demonstrations and only the four 1-min trials of the dog. The inter-coder agreement (weighted Cohen’s Kappa) was excellent for the irrelevant-action scores and the relevant-action scores (0.95 each).

How well a dog learns from watching a human demonstrator can be influenced by breed type^26^. Therefore, we also clustered breeds into “cooperative” and “independent” working-dog types, as in Dobos and Pongrácz^26^, and “other” for non-working line and mixed breed dogs [see Participant List, Supplementary Materials]. For example, a border collie was a “cooperative” breed-type, and a border terrier was an “independent” breed-type.

#### Data analysis

The data was analysed in R (Version 4.3.0^27^) using the software RStudio. To examine whether prior experience influenced copying behaviour in dogs, we fitted two ordinal (i.e., cumulative logit link) mixed models: one with the irrelevant-action copying scores (IRR, Eq. 1) and one with the relevant-action copying scores (REL, Eq. 2) as response variables. Ordinal mixed models were built using the function ‘clmm’ in the package ‘ordinal’ (Version 2022.11-16^28^). The IRR model (Eq. 1) included experimental condition (prior experience and no-prior experience), trial (1, 2, 3, 4), and irrelevant-action timing (before the goal and after the goal) as the test predictors of interest. We also added breed type as a control predictor. Our reference categories were condition: no-prior experience, timing: before, and breed: other. The REL model (Eq. 2) was identical, but did not contain irrelevant-action timing (before the goal and after the goal) as a predictor. Subject ID was included as a random intercept effect to account for repeated measurements of the same individuals, and trial within subject was included as a random slope effect^29,30^. Trial was z-transformed to ease model convergence and acquire more easily interpretable model coefficients^31^.

Our variables and predictors matched our pre-registration of the analysis for both models (Eqs. 1 and 2), however, we decided (1) to remove the control group from the main model for cleaner interpretability and model simplicity, (2) to conduct baseline comparisons in separate secondary analyses, and (3) to includ a three-way interaction in the IRR model (condition * trial * timing), as irrelevant-action timing was also expected to vary depending on prior experience. The two *full* ordinal mixed models were as follows:

1 $${\text{IRR }}\sim {\text{ condition }}\left( {{\text{no-prior}}\;{\text{ experience}},\;{\text{ prior }}\;{\text{experience}}} \right) \, *{\text{ z}}.{\text{trial }}*{\text{ timing }}\left( {{\text{before}},\;{\text{ after}}} \right) \, + {\text{ breed }}\left( {{\text{cooperative}},\;{\text{ independent}},\;{\text{ other}}} \right) \, + \, \left( {{1 } + {\text{ z}}.{\text{trial}}|{\text{subject ID}}} \right)$$

2 $${\text{REL }}\sim {\text{ condition }}\left( {{\text{no-prior}}\;{\text{ experience}},\;{\text{ prior}}\;{\text{ experience}}} \right) \, *{\text{ z}}.{\text{trial }} + {\text{ breed }}\left( {{\text{cooperative}},\;{\text{ independent}},\;{\text{ other}}} \right) \, + \, \left( {{1 } + {\text{ z}}.{\text{trial}}|{\text{subject ID}}} \right)$$

And our two *null* ordinal mixed models (without the test predictors of interest) were as follows:

3 $${\text{IRR }}\sim {\text{ breed }}\left( {{\text{cooperative}},\;{\text{ independent}},\;{\text{ other}}} \right) \, + \, \left( {{1 } + {\text{ z}}.{\text{trial}}|{\text{subject ID}}} \right)$$

4 $${\text{REL }}\sim {\text{ breed }}\left( {{\text{cooperative}},\;{\text{ independent}},\;{\text{ other}}} \right) \, + \, \left( {{1 } + {\text{ z}}.{\text{trial}}|{\text{subject ID}}} \right)$$

Absence of collinearity was verified by calculating the Variance Inflation Factor (VIF) for a corresponding linear mixed model using the R package “car” version 3.0-12^32^. The full models had VIFs all less than ‘2’ (maximum VIF IRR model: 1.94, maximum VIF REL model: 1.38). Model stability was assessed by comparing the estimates from the models including all data with estimates obtained from models in which individuals were excluded one at a time^33^. This revealed that both models were of good stability. To avoid ‘cryptic multiple testing’^34^, we compared the full models to null models without the test predictor(s) of interest (Eqs. 3 and 4). If this showed a significant effect, we continued to test the individual predictors’ effects. We determined the significance of individual predictors by dropping them from each of the full models, one at a time using R’s ‘drop1’ function, and comparing the resulting model with the corresponding full model. For model comparisons, we used likelihood ratio tests^35^ and started with the highest order (three-way) interaction. If higher-order interactions did not show significance, we iteratively removed them from the model. Thus, we could reliably interpret any lower order terms in the model^36^. We calculated 95% confidence intervals for the model estimates and fitted values by applying the ‘bootMer’ function of the package ‘lme4’, applying 1,000 parametric bootstraps. Estimated marginal means and standard deviations were calculated in R with the ‘emmeans’ package (Version 1.10.3^37^) for the IRR ordinal mixed model, in order to visualise the model’s results.

Control group comparisons, henceforth, “baseline comparisons”, were conducted of the irrelevant-action scores and relevant-action scores using the condition’s means and standard deviations for t-tests (‘t.test’ function in R). These analyses were conducted to confirm whether the irrelevant-action scores were indeed socially facilitated by the demonstration. We used Bonferroni correction for multiple testing on the t-tests’ corresponding *p*-values.

### Informed consent

Informed consent was obtained from the dogs’ caregivers, who participated voluntarily with their dogs and were told that they could withdraw from the study at any time. Extra informed consent was also obtained to publish any identifying information and images.

## Results

### Descriptive overview

Overall, overimitation frequency was modest in the box-stepping overimitation task. Each dog had two opportunities to score in the irrelevant action per trial, *before* and *after* the goal. For the irrelevant action, dogs with prior experience scored 1 + in 77/200 instances (dogs N = 25, trials N = 100), dogs without prior experience scored 1 + in 86/224 instances (dogs N = 28, trials N = 112), and control dogs scored 1 + in 48/224 instances (dogs N = 28, trials N = 112). Each dog had one opportunity to score in the relevant action per trial. For the relevant action, dogs with prior experience scored 1 + in 97/100 trials, dogs without prior experience scored 1 + in 110/112 trials, and control dogs scored 1 + in 81/112 trials. Table 3 displays the trial frequencies of both irrelevant and relevant scores per condition.

**Table 3:** Trial frequencies of the irrelevant- and relevant-action scores for each condition, with the irrelevant-action scores split between before and after (timing).

Score	With prior experience (N = 100 trials)	Without prior experience (N = 112 trials)	Control (N = 112 trials)
Irrelevant action (before)			
0	53	46	72
1	28	21	24
2	12	31	10
3	1	7	1
4	6	7	5
Irrelevant action (after)			
0	70	92	104
1	17	16	8
2	7	3	0
3	3	1	0
4	3	0	0
Relevant action			
0	3	2	31
1	2	11	14
2	13	22	16
3	59	49	40
4	23	28	11

There were four trials per dog.

### Irrelevant-action copying (IRR ordinal mixed model)

**Fig. 2:**
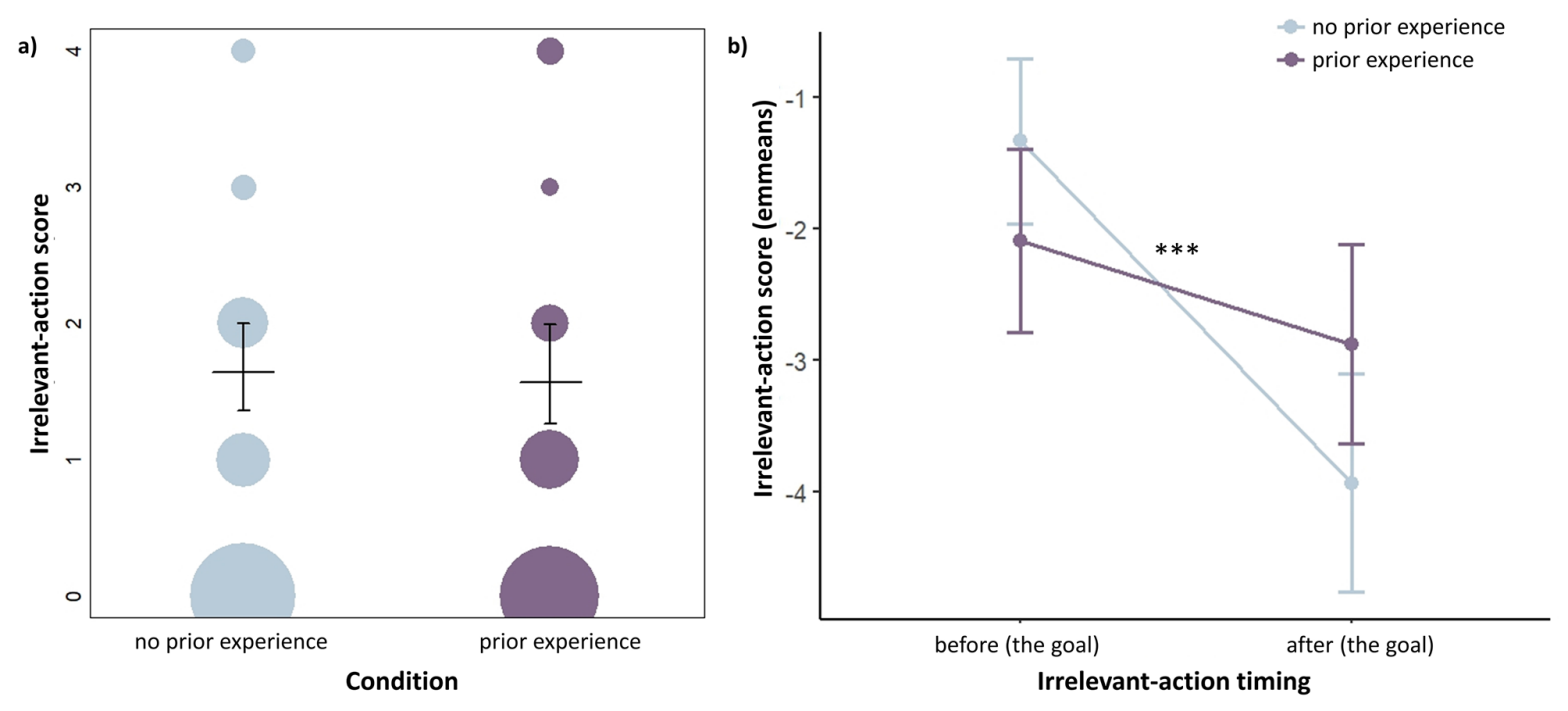
The final IRR mixed model’s results: (**a**) the irrelevant-action scores per condition, with circle size depicting scoring frequency (i.e., larger circle = larger number of scores at that level), and the model’s confidence interval fitted values and upper/lower limits as line segments and bars; (**b**) the irrelevant-action score’s estimated marginal means for the interaction (condition:timing), calculated from the model are also shown, with ‘***’ representing a significant *p*-value of < .001.

The ordinal mixed model revealed that dogs who had prior experience of the task had a higher likelihood of scoring in the irrelevant-action *after* the task’s goal (scoring 3 + in the relevant action), and those without prior experience had a higher likelihood of scoring in the irrelevant-action *before* the task’s goal (Fig. 2). There were also significant effects of timing and trial in the model, where all dogs scored in the irrelevant action less per trial, and less after the goal. Therefore, prior experience influenced when, not whether, dogs copied the irrelevant action.

In detail, the results showed a significant full-null model comparison for the IRR ordinal mixed model (χ^2^ =  − 84.908, df = 7, *p* < 0.001), meaning that our test predictors (condition, z.trial and timing) had an effect on irrelevant-action scores. The full model’s three-way interaction was not significant (χ^2^ = 2.89, df = 2, *p* = 0.236), and was therefore removed from the model to form a reduced model with only two-way interactions. Our likelihood ratio tests of the final, reduced model had one significant interaction of condition and timing (χ^2^ = 15.895, df = 1, *p* < 0.001), a significant effect of z.trial (χ^2^ = 9.855, df = 1, *p* = 0.002), but a non-significant effect of breed type (χ^2^ = 1.847, df = 2, *p* = 0.397).

On further inspection of the final model’s coefficients, we found the following significant effects and significant interaction on irrelevant-action scores: timing: after (Z =  − 7.39, *p* < 0.001), z.trial (Z =  − 3.092, *p* = 0.002), and an interaction of the prior experience condition and timing: after (Z = 3.913, *p* < 0.001). The final IRR model’s output is available in Supplementary Materials, Appendix 1.

### Relevant-action copying (REL ordinal mixed model)

Results of the REL ordinal mixed model showed a non-significant full-null model comparison for the IRR ordinal mixed model (χ^2^ = 3.218, df = 3, *p* = 3.592), meaning that the test predictors (condition and z.trial) did not have an effect on relevant-action scores. The REL ordinal mixed model’s output can be found in Supplementary Materials, Appendix 1.

### Baseline (control group) comparisons

#### Irrelevant-action copying (before the goal and after the goal) compared to the control group

Compared to dogs who did not have task demonstrations nor task prior experience (control group), dogs without (but not with) task prior experience scored significantly higher in the irrelevant action *before* the goal (scoring 3 + in the relevant action, prior experience: t(202.31) =  − 1.32, *p* = 0.188; no-prior experience: t(214.6) =  − 3.89, *p* < 0.001). Dogs both with and without task prior experience scored significantly higher than the control group in the irrelevant action *after* the goal (scoring 3 + in the relevant action, prior experience: t(111.6) =  − 4.49, *p* < 0.001; no-prior experience: t(160.71) =  − 2.72, *p* = 0.015). To control for multiple comparisons, Bonferroni corrections were applied to all four *p*-values from the baseline comparisons of irrelevant-action scores.

**Fig. 3:**
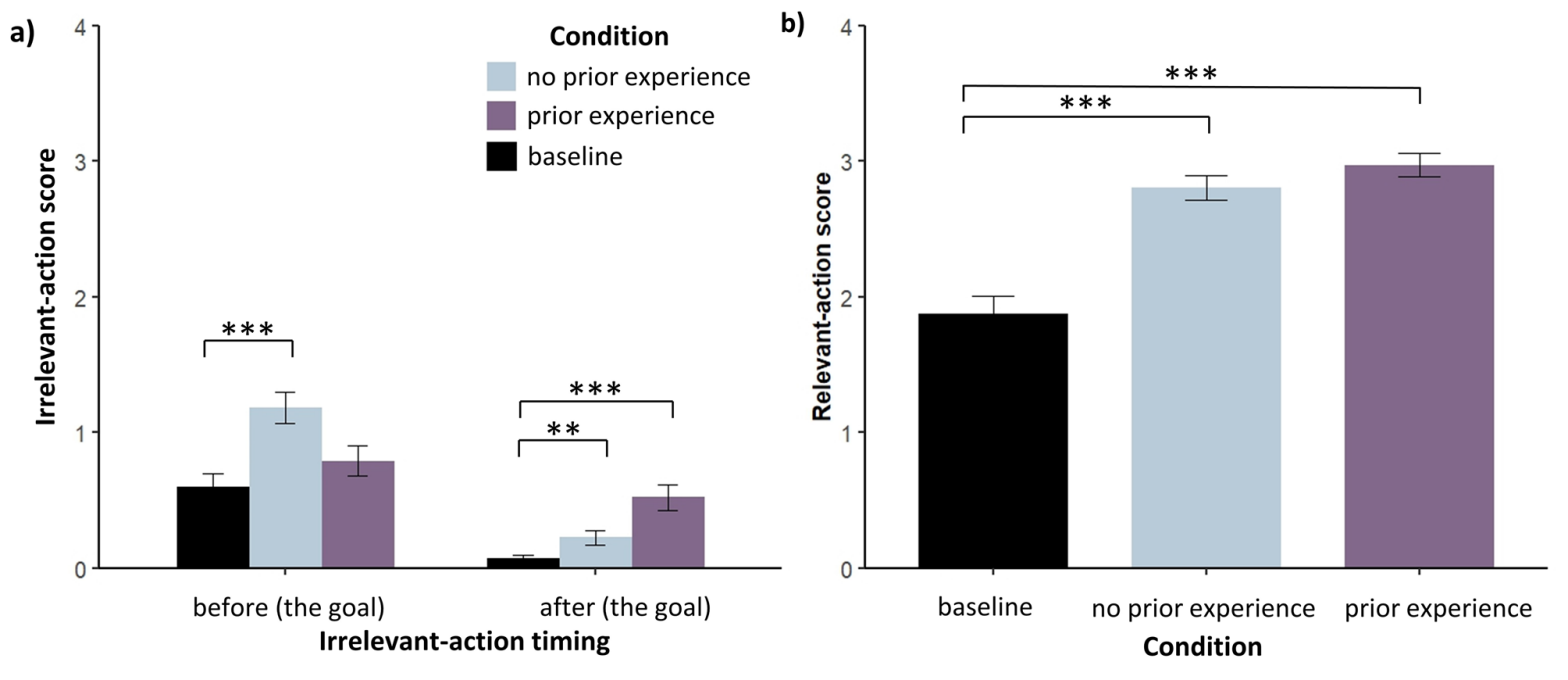
Baseline (control group) comparison barplots for: (**a**) irrelevant-action scores, (**b**) relevant-action scores. The horizontal lines denote a statistically significant t-test comparison (Bonferroni corrected) between groups, with ‘***’ representing a *p*-value < .001 and ‘**’ representing a *p*-value < .01.

The mean irrelevant-action scores *before* the goal were: 0.60 for the control group (baseline), 0.79 for the prior experience group, and 1.18 for the no-prior experience group of dogs. The mean irrelevant-action scores *after* the goal were: 0. 07 for the control group (baseline), 0.52 for the prior experience group, and 0.22 for the no-prior experience group of dogs (Fig. 3).

#### Relevant-action copying compared to the control group

Compared to dogs who did not have a task demonstration nor task prior experience (control group), dogs with and without task prior experience scored significantly higher in the relevant action (prior experience: t(184.99) =  − 6.94, *p* < 0.001; no-prior experience: t(198.6) =  − 5.72, *p* < 0.001). To control for multiple comparisons, Bonferroni corrections were applied to the two *p*-values from the baseline comparisons of relevant-action scores.

The mean relevant-action scores were: 1.88 for the control group (baseline), 2.97 for the prior experience group, and 2.80 for the no-prior experience group of dogs (Fig. 3).

## Discussion

In summary, dogs with and without prior experience did not differ in their overimitation frequency (irrelevant-action copying), but differed in their overimitation *timing* in relation to the task goal (accessing a toy-ball from a bucket). Dogs who did not have prior experience of our task were copying the irrelevant-action *before* the task goal, while dogs who had prior experience were copying the irrelevant-action *after* the task goal. These latter dogs already had understanding of the actions’ consequences, yet, still overimitated. Dogs without prior experience did the opposite—implying a degree of causal misunderstanding. Baseline (control group) comparisons confirmed that these results were facilitated by the cargiver's social demonstration. We also found that irrelevant-action scores decreased with each trial and were mostly before the goal. For our relevant-action scores, there was no difference between dogs with and without prior experience, however both groups with social demonstrations had significantly higher scores than our no-demonstration control group. Overall, there were less dogs in our box-stepping overimitation task who were copying our irrelevant action than dogs in the tasks of previous studies (i.e.^8,9,21^).

Prior experience did not lead dogs to overimitate less than dogs without prior experience. This result differed from that of humans, as task experience led to a reduction of overimitation. It is important to note, however, that human overimitation is usually recorded *before* a goal, unless otherwise specified (such as in^38^). And studies have shown that children will still delay their goal and overimitate a demonstrator, even with causal understanding of the task at hand^16–18,23^. Hypothetically, if we only recorded overimitation *before* the goal, then dogs with prior experience would have had reduced overimitation levels too. But these dogs were instead prioritising the task goal, not reducing their overimitation. Unlike dogs without prior experience, dogs with it were copying the demonstrator’s irrelevant action later, once the (physical) goal was satisfied. Dogs who overimitated later had their reward ball fully available (sometimes still in their mouth), so the irrelevant box obviously had no effect on the already-obtained reward. This post-goal overimitation resembles a “reward (me) now, copy (you) later” approach, which contrasts the “copy now, refine later” approach said to be used by humans^14^. Perhaps humans are more sensitive to action order and how things should conventionally be done, while (task) knowledgeable dogs have no conventional motive to *not* go immediately to their reward, especially if they have not been commanded otherwise.

With task experience from trials, dogs learned of the consequences of the actions and reduced their overimitation. Each trial offered the opportunity for dogs to learn the functions of the irrelevant and relevant actions. If they successfully obtained the ball, then they would have learned that only opening the bucket led to the reward and the box-stepping did not. In contrast, dogs in Mackie and Huber’s^21^ task did not overimitate less per trial. Since most of their dogs overimitated after the goal and most of our dogs overimitated before the goal, then perhaps the dot-touching action was more obviously irrelevant than our box-stepping action. Thus, it is likely that the (mostly post-goal) overimitation in Mackie and Huber’s study was motivated by potential social rewards, while the “*pre-goal*” overimitation (overimitation before the goal) in our study could be explained by elements of causal misunderstanding. Post-goal overimitators, in both studies, seem to be the dogs who were able to separate the task into two goals; a goal to efficiently obtain the physical reward and a goal to copy and be-like their demonstrator. This secondary goal for overimitation in dogs needs further investigation.

There may be a case in which prior experience not only provided dogs with understanding of the actions’ consequences, but also extra motivation. The task goal and how to achieve it became clearer if experience was gained. This could have made our dogs more focused on the physical goal, especially because they were positively reinforced with the ball reward if they successfully opened the bucket. Thus, a stronger motivation would have tugged them towards the relevant action first (and not the non-rewarding, unreinforced irrelevant action). However, motivation *alone* is not enough to explain this differences in timing for dogs with and without prior experience. All of our dogs, including the control group, were reinforced to retrieve the ball from their game of fetch, and they all saw the location of the ball in the item habituation phase. Yet, we still found an effect of prior experience (irrespective of trial). Dogs with prior experience, but not without, must have indeed gained appropriate knowledge of action consequences to clarify which action was the relevant one. Expertise (from task experience) then fuelled the dogs’ motivation to obtain the reward as soon as possible.

Together with our finding that dogs without prior experience overimitated more often before the goal, our results suggest that *both* (some sort of) causal misunderstanding and non-instrumental (social) motivations were at play in this study. Dogs in the process of learning a task may copy irrelevant demonstrations because the actions’ consequences are ambiguous, while dogs who have already learned the task copy irrelevant demonstrations non-instrumentally. Of course, some dogs with prior experience and/or trial experience still overimitated beforehand, but this was a rather rare occurrence. These particular dogs may have had such high motivation to copy their caregiver that they were willing to delay their reward to match the observed action order, in this context. Studies have shown that dogs are able to delay their reward longer than wolves^39^, delay copying humans^40^, and delay the pressing of a button to wait for their partner a cooperative task^41^. Dogs have even followed a human’s misleading point towards an empty food bucket when they knew where the food actually was^42^. It is not entirely surprising that at least some dogs would also go as far as inhibiting their reward (that they already knew how to get) in order to follow their caregiver’s irrelevant demonstration, however dogs with prior experience overall did not interact with the irrelevant box before the goal significantly more than the baseline level. Meanwhile, the overimitators who were unable to inhibit the reward copied their caregiver after they had already satisfied the physical goal. A noteworthy mention here is that both dogs with and without prior experience engaged in significantly more post-goal irrelevant-action scoring than our control group, in which there would have been little social motivation for the task given that there were no caregiver demonstrations.

The present study was able to link pre-goal overimitation to causal misunderstanding, and post-goal overimitation to non-instrumental (social) drives in dogs. However, there are still many avenues to explore. For example, what type of irrelevant-actions are dogs more likely to copy? Dogs have been shown to be sensitive to transitive and intransitive actions; whether or not actions involve an object. Actions that are intransitive are supposedly harder for dogs to copy (from another dog in^43^,from a human in^6^). In human overimitation studies, “pseudo-instrumental” irrelevant actions, ones in contact with the reward container, are more likely to be copied than those which are physically detached^44^. This is in accordance with the “contact principle”, which claims that children often use physical contact to determine causal connections (i.e.^14^). Johnston et al.^9^ connected-but-irrelevant lever may have elicited more copying than our disconnected box-stepping, if the contact principle applies to dogs—but this is yet to be investigated.

What is also uncertain is the exact non-instrumental motivation dogs have when they copy an irrelevant action while knowing that it does not lead to a physical reward. Dogs seem to overimitate their closely-bonded caregiver more than strangers^10,11^, but is this to please their caregiver? Is it a social game? Or is it really because they want to be-like their affiliated caregiver? Context manipulation through demonstration style could help determine dogs’ secondary goal. For example, Hoehl et al.^44^ found differences in child overimitation when the demonstration was in a communicative or non-communicative style using pedagogical cues. For dogs, one could manipulate the ostensive cues used in the demonstration, such as eye-contact and verbalisations. This could tell us if dogs view the task as a sort of training session from the caregiver, something they *have* to do. Now that we have provided evidence that dogs have motives unrelated to causal misunderstanding, we recommend future research to explore the social components that may facilitate post-goal overimitation.

A potential limitation of the present study was that instances of perfect overimitation were overall low. This is likely because our box-stepping irrelevant action was more demanding than knocking a lever or touching a dot. It required the lifting of at least one paw to place into a novel box, which may have been physically and mentally effortful. In some trials, dogs even lifted their paw *as if* they were to step into the 12 cm-high box, but then they lowered their paw again. Perhaps with more trials and item habituation, those dogs would have also overimitated their caregiver. Another limitation is that stepping into an object, as an action, may have been experienced during the dogs’ lifetime. This problem is not unlike that which human children overimitation studies face: imitation tasks contain a novel context, novel objects, and novel combinations of actions, but irrelevant actions themselves such as clapping or lever-moving (i.e.^16^) likely would have been experienced by 5-year-old children in their day-to-day lives. Our task’s actions were considered novel for dogs in the context of the task, however, half of our study’s dogs (46/81) did have some history of crate training, e.g., a command to get into a crate for car rides or bedtime, according to their caregivers. Nonetheless, our conditions were closely balanced also for any crate-training history (14/25 prior experience group, 17/28 no-prior experience group, 14/28 control group). Despite crate training history, there was still some hesitation from dogs to step into the irrelevant box in our box-stepping overimitation task. In any case, while our box-stepping action may have been a limitation of the present study, utilising a new task and new actions had advantages in terms of contextual variety and ecological validity in the literature of overimitation in dogs. We would encourage future studies on this topic to do the same.

### Conclusion

In conclusion, this study’s findings suggest that domestic dogs overimitate in two distinct ways; with (“causal”) misunderstandings and with social motivations. Dogs did not overimitate *less* if they had prior experience of the task, only *later*. By measuring the timing of overimitation, before (pre-goal) and after the goal (post-goal), we were able to find that prior experience influenced when, and not whether, dogs would copy the causally-irrelevant action. Dogs prioritised obtaining the ball if they already knew how to solve the task in a “reward (me) now, copy (you) later” approach. However, a direct comparison of overimitation timing with humans is necessary to understand if this approach is indeed partly what differentiates dog overimitation from that of humans.

## Supplementary Information

Supplementary Information.

## Data Availability

Data and code are available on the Open Science Framework (OSF) through the following link: https://osf.io/vx2ph/.
