# Supplementary material for: Dogs with prior experience of a task still overimitate their caregiver
Source: Sci Rep. 2024 Sep 6;14:20806. doi: 10.1038/s41598-024-70700-3 (PMC11379846; doi:10.1038/s41598-024-70700-3)
Supplement: Supplementary file 1 — Supplementary Information. [file 41598_2024_70700_MOESM1_ESM.docx]

**Dogs with prior experience of a task still overimitate their caregiver:**

**Supplementary Materials/Appendix**

Louise Mackie^1*^ and Ludwig Huber^1^

^1^Comparative Cognition, Messerli Research Institute, Department of Interdisciplinary Life Sciences, University of Veterinary Medicine Vienna, Vienna, Austria

***Correspondence:**Louise Mackie
[Louise.Mackie@vetmeduni.ac.at](mailto:Louise.Mackie@vetmeduni.ac.at)

**Keywords**: domestic dogs, prior experience, copying, social learning, causal understanding

**Pre-registration**: <https://aspredicted.org/Y73_VFL>

# Abstract

Domestic dogs have been shown to copy their caregiver’s actions, including ones which are causally-irrelevant to a physical goal – a behaviour called “overimitation”. In a new overimitation task with a non-food reward, this study investigated “causal misunderstanding” – falsely assuming causally-irrelevant actions to have functional relevancy – as an explanation for dog overimitation (N = 81). By providing dogs with prior experience of the task to learn about the consequences of its irrelevant box-stepping and relevant bucket-opening action to obtain a toy-ball, we tested whether and when dogs would copy their caregiver’s irrelevant-action demonstrations. Dogs with and without prior experience were compared to a third (control) group of dogs, who had neither prior experience nor caregiver demonstrations of the task. Results revealed that the timing of overimitation, rather than its frequency, was closely related to dogs' prior experience: dogs with prior experience attended to their reward first, then interacted with the irrelevant box later (“post-goal overimitation”), while dogs without prior experience first interacted with the irrelevant box (“pre-goal overimitation”). Our results suggest that, when action consequences are understood, dogs are overimitating for a secondary social goal that is clearly distinct from the task’s goal to obtain a physical reward.

# Supplementary Materials

Participant List (excel file), experimental instructions (English/German), and the dog’s task trial video example are all available on OSF with the following link: <https://osf.io/vx2ph/?view_only=389a5556e706445088ddc097a063f2da>

# Appendix

| **Appendix 1.** Table output of the two ordinal mixed models for irrelevant-action (significant full-null model comparison) and relevant-action scores (non-significant full-null model comparison). | | | | | | |
| --- | --- | --- | --- | --- | --- | --- |
| **Term (effect)^(1)^** | **Estimate** | ***SE*** | **Lower CI^(3)^** | **Upper CI^(3)^** | ***Z-value*** | ***P-value*^(4)^** |
| **Irrelevant-action scores** | | | | | | |
| 0\|1 | -0.644 | 0.497 | -1.296 | -1.635 | 0.320 | ^(5)^ |
| 1\|2 | 0.737 | 0.496 | 1.487 | -0.178 | 1.747 | ^(5)^ |
| 2\|3 | 2.256 | 0.516 | 4.372 | 1.319 | 3.415 | ^(5)^ |
| 3\|4 | 2.930 | 0.544 | 5.389 | 1.974 | 4.248 | ^(5)^ |
| conditionpre-exp | -0.759 | 0.445 | -1.706 | -1.703 | 0.089 | 0.088 |
| **timingafter** | **-2.595** | **0.351** | **-7.390** | **-3.406** | **-1.950** | **<0.001***** |
| **z.trial** | **-0.387** | **0.125** | **-3.092** | **-0.662** | **-0.163** | **0.002**** |
| breedCooperative | -0.352 | 0.524 | -0.671 | -1.413 | 0.758 | 0.502 |
| breedIndependent | 0.298 | 0.610 | 0.488 | -0.912 | 1.506 | 0.626 |
| **conditionpre-exp:timingafter** | **1.812** | **0.463** | **3.913** | **0.954** | **2.775** | **<0.001***** |
| **Relevant-action scores** | | | | | | |
| 0\|1 | -6.603 | 1.581 | -4.177 | -15.041 | -4.228 | ^(5)^ |
| 1\|2 | -4.025 | 1.333 | -3.019 | -11.134 | -1.751 | ^(5)^ |
| 2\|3 | -0.514 | 1.205 | -0.427 | -4.737 | 2.097 | ^(5)^ |
| 3\|4 | 5.741 | 1.418 | 4.049 | 3.481 | 11.569 | ^(5)^ |
| conditionpre-exp | 0.465 | 1.019 | 0.456 | -1.648 | 2.919 | 0.648 |
| z.trial | 0.176 | 0.390 | 0.452 | -0.675 | 1.060 | 0.652 |
| **breedCooperative** | **2.925** | **1.351** | **2.164** | **0.378** | **7.012** | **0.030*** |
| breedIndependent | 3.036 | 1.583 | 1.918 | -0.079 | 7.769 | 0.055 |
| conditionpre-exp:z.trial | -0.867 | 0.567 | -1.529 | -2.296 | 0.400 | 0.126 |
| (1) Condition, timing, and breed had ‘**condition: no-pre-exp’**, ‘**timing: before’**, and ‘**breed: other’** as the reference categories respectively.  (2) Trial number was z-transformed (z.trial) to a mean of zero and a standard deviation of one, mean (sd) of trial number was 2.5 (1.19)  (3) Confidence intervals (CI) were calculated with 1,000 parametric bootstraps in R.  (4) Signif. codes: p-values of <0.001 are ‘***’, <0.01 are ‘**’, and <0.05 are ‘*’, and in bold  (5) Not indicated because of having a very limited interpretation | | | | | | |
